# Digital Metrology for Nanoindentation: Synthetic Data Generator for Error Identification

**DOI:** 10.3390/mi16121394

**Published:** 2025-12-11

**Authors:** Giacomo Maculotti, Lorenzo Giorio, Gianfranco Genta, Maurizio Galetto

**Affiliations:** 1 Department of Management and Production Engineering, Politecnico di Torino, Corso Duca degli Abruzzi 24, 10129 Turin, Italy; gianfranco.genta@polito.it (G.G.); maurizio.galetto@polito.it (M.G.); 2 Department of Applied Science and Technology, Politecnico di Torino, Corso Duca degli Abruzzi 24, 10129 Turin, Italy; lorenzo.giorio@polito.it

**Keywords:** nanoindentation, digital twin, synthetic dataset, quality control

## Abstract

Digital metrology enables precise, real-time measurement and data analysis using digital tools, which enhances accuracy and efficiency in manufacturing and quality control. Among key enabling technologies, Digital Twins allow continuous control, enabling predictive maintenance, faster error detection, and optimised performance of the measurement system. A current challenge is establishing traceability for the Digital Twins and for the data processing algorithms implemented in digital metrology. Nanoindentation is a challenging measurement technique that may be susceptible to several random and systematic measurement errors. This work presents a parametric synthetic dataset generator for quasi-static, room-temperature nanoindentation that incorporates correlation and covariance among simulated quantities. The method models indentation responses through a power-law formulation fitted via Orthogonal Distance Regression, allowing for traceable and physics-informed datasets. The generator enables the association of uncertainty with simulated results, supporting its use within a metrological framework. Its performance is benchmarked against non-parametric methods such as bootstrapping, showing comparable accuracy with significantly reduced computational cost and improved representativeness. Furthermore, the methodology can simulate main measurement errors for advanced material characterisation and develops a traceable tool based on synthetic data which could be used to train advanced quality control tools for the detection of main measurement errors.

## 1. Introduction

By means of Artificial Intelligence (AI) and Machine Learning (ML), digital transformation can achieve higher performances, effectiveness, and increased sustainability by improving manufacturing processes to reduce waste and defects [1,2]. In fact, AI and ML allow enhanced condition monitoring [3], early fault diagnosis, and reliable predictive maintenance [4], fostering goals of zero-defect and zero-waste manufacturing [5,6]. Among the others, Digital Twins (DT) [7,8] have been established as an effective enabling technology for digital transformation to describe, monitor, predict and control [9,10] manufacturing elements, processes, and systems [11,12]. Increasingly, ML approaches and AI are being used to model DTs of physical entities, for such data-driven approaches [13] can overcome expensive modelling efforts required by physics-based and analytical modelling strategies [14].

However, AI and ML severely depend on the quality of the data. Within this framework, data metrology [15] and virtual metrology [16] are gaining a critical and core role to establish trustworthiness of advanced modelling tools. Specifically, data metrology aims at guaranteeing the quality and traceability of data, while providing all relevant information to quantify uncertainty for the particular application [16]. Furthermore, a critical challenge for digital transformation is the availability of big data, which is required for the application of AI and ML to state-of-the-art monitoring approaches, e.g., DTs [17,18]. Also, traceable virtual experiments and digital twins can only be achieved when trustworthy data are available [19].

Accordingly, synthetic datasets are increasingly resorted to augment the representativeness and the numerosity of the training dataset. In fact, rare or extreme conditions can often be underrepresented due to their low occurrence, e.g., due to failures, or extreme costs and risk of related experiments. To overcome such limitations, synthetic data generation has emerged as a strategic solution. In fact, synthetic data generation consists of creating artificial information that accurately reflects the physical and statistical characteristics of real processes. The technique has evolved along a path of increasing sophistication and has found several applications in current industrial and academic applied research.

Synthetic data can be generated mainly by analytical models, data-driven approaches, or by a hybrid method [20]. Analytical models leverage closed-form solutions based on the physics constitutive and descriptive equations of the considered phenomenon. Although highly elegant and computationally effective, they often rely on simplifying assumptions, which might significantly bias the estimated response. Finite element method (FEM) simulations enable overcoming simplifying assumptions by iteratively reaching numerical solutions, albeit at the cost of introducing a large number of parameters, high computational costs, and a cost-efficiency that drives the approximation and bias of the response. Data-driven approaches stand on the opposite side of modelling. In fact, data-driven approaches build models starting from available data without prior knowledge of the system physics. Machine Learning has made data-driven approaches widely used [21,22], e.g., by exploiting generative adversarial networks [23], reinforcement learning, variational autoencoders, Markov chain models, and Gaussian process regressions [21,22]. Inverse measurement problems solutions try to combine data-driven methods, most typically Bayesian metaheuristics, with numerical simulation, to find a set of hyperparameters that minimises the bias of the simulation [24,25,26]. However, the solution is computationally and experimentally extremely expensive, potentially requiring thousands of indentations in different conditions, and may be not unique [27]. Lastly, hybrid approaches aim to combine the simplicity and potential of ML with the prior knowledge typical of analytical methods. This is typically obtained by constraining some parameters and by defining the analytical form of the model in agreement with the physics of the system [28].

Examples of synthetic data generation can be found both for modelling complex systems and processes and for advanced quality controls.

As far as the complex systems case is concerned, for example, Toro et al. developed a synthetic data generator for smart measurement sensors of electrical quantities integral to advanced grid infrastructures. The generator was based on an analytical model and was required due to the scarcity of real-world data because of privacy, security, and grid accessibility [29]. Urgo et al. exploited synthetic image generation via virtual reality tools to augment the training dataset for a manufacturing system quality control that tracks objects on the production line [3,4]. Lopes et al. exploited synthetic data generated by a Random Graph model to train a DT of a production system, incorporating rare scenarios such as those related to failures, and to tune the response on different bottleneck, allocation, and productivity scenarios otherwise impossible or extremely expensive to obtain experimentally [30].

Examples of effective use of synthetic datasets to model and control manufacturing processes can be found in Kim et al., who leveraged synthetic data generation to improve the robustness of DT training for a pick-and-place operation by a collaborative robot within a human–machine interaction framework. Such a scenario requires both object detection and gripping, as well as obstacle avoidance, which, for robust training, requires big data collected in a safe environment. Graphic rendering simulations were leveraged to generate synthetic images used to train the object detection and gripping optimisation, while virtual reality was exploited to simulate obstacles and robot response optimisation by means of a reinforcement learning strategy [31]. Loaldi et al. overcame experimental costs to model the complex interaction of process parameters and part quality in micro-injection moulding processes by synthetic data generation through traceable FEM simulations [32]. Similarly, Solis-Rios et al. reduced the cost of investigating the relationship between process parameters for PE-Oxides nanofibres by Neural Network generation of synthetic data [33].

Similarly, measurement and quality controls largely benefited from synthetic data. For example, Nguyen et al. generated synthetic data for automotive wiring to compensate for the cost of acquiring real data by a neural network for geometrical data and by FEM simulation for electrical functionality [34]. Synthetic data generation by generative artificial networks has also been largely exploited to increase the training datasets for Machine Vision applications, e.g., for surveillance [35], and for visual inspection of quality of welds [36] or packaging of microelectronics [37].

Additionally, widespread adoption of synthetic dataset generation can be found for metrological applications to improve the accuracy and measurement uncertainty of measurement techniques. Lafon et al. developed a methodology to generate reference data to benchmark the performance of registration algorithms [38].

Extensive use can also be found in surface science to support nanometrology and increase the informativeness of characterisation techniques. Necas and Klapetek recently reviewed the use of synthetic data for nanometrology of scanning probe microscopy (SPM), highlighting benefits both in terms of accuracy, uncertainty, and measurement duration. Also, synthetic data allows compiling an atlas of measurement error useful both for training and measurement compensations [39]. Advanced deep learning modelling of SPM-based nanoindentation was obtained by synthetic data generation based on contact models [40].

Among the other surface characterisation techniques, nanoindentation [41,42] has largely been the object of data fusion through synthetic data generation. This work aims at developing a metrological framework for synthetic data generation to support error detection and modelling in nanoindentation. The following subsection will briefly introduce the fundamentals of nanoindentation, review state-of-the-art applications of synthetic data to nanoindentation, and define the scope of the work.

### 1.1. Fundamentals of Nanoindentation

Nanoindentation, i.e., Instrumented Indentation Test in the nano-range [42], is a depth-sensing, non-conventional hardness measuring technique which allows high-resolution characterisation of mechanical properties of surface layers [41,43]. It allows evaluating estimates of Young modulus, creep, and relaxation behaviour of materials [42,43] and coatings [44,45,46]. It finds extensive applications, e.g., to study grain and phase size, distribution, and mechanical properties [47], to characterise properties’ gradient in functional coatings [46,48], directional manufacturing [47], and finishing processes [49], and it can also measure film thickness [50]. Most innovative applications are now focusing on in-operando characterisation, typically for high-temperature aerospace environments [51] and for biological materials [52].

Nanoindentation is performed by applying a loading–holding–unloading force cycle to a sample by means of an indenter. The (most typically) applied force *F* and the indenter penetration depth *h* in the sample are measured during the whole experiment, thus obtaining the Indentation Curve (IC), i.e., *F*(*h*). The characterisation at nanoscales is enabled by the calibrated correlation between the area of the contact surface of the indenter with the sample and the penetration depth, i.e., the area shape function *A*(*h*).

Traceability is obtained by calibrating the force and displacement scales, and by calibrating the area shape function parameters and the frame compliance *C_f_*, needed to compensate for the elastic deformation of the indentation platform occurring during the load application. In particular, the corrected contact depth *h_c_* is obtained as in Equation (1a), and the maximum corrected contact depth *h_c,max_* results in Equation (1b):

(1a) $$h_{c}=h-h_{0}-C_{f}F$$

(1b) $$h_{c,max}=h-h_{0}-C_{f}F_{max}-\epsilon \left({\frac{1}{S}}_{m}-C_{f}\right)F_{max}$$

where *h*_0_ is the zero contact point, $C_{f}F$ accounts for the elastic deformation of the indentation testing machine, and $\epsilon \left({\frac{1}{S}}_{m}-C_{f}\right)F_{max}$ for the elastic deformation of the sample at the onset of the unloading [42,53]. The latter term includes a correction factor to cater for the indenter geometry, i.e., ϵ, and for the measured contact stiffness, *S_m_*. *S_m_* is evaluated as the derivative of the IC at the onset of the unloading. The derivative evaluation requires modelling the *F*(*h*) relationship [42]. Such a task, although other approximations have been suggested [54,55], is best obtained by leveraging Sneddon’s solution of Boussinesq’s contact problem [56]. This assumes a power-law relationship, see Equation (2), where the parameters depend on the material of the sample and the indenter and on the indenter geometry.

(2) $$F={\beta_{0}\left(h_{c}-\beta_{1}\right)}^{\beta_{2}}$$

Two different models, i.e., sets of parameters, can be obtained by studying the loading and unloading segment of the IC, respectively, with parameters $\left\{\beta_{0,l},\;\beta_{1,l},\beta_{2,l}\right\}$, and $\left\{\beta_{0,u},\;\beta_{1,u},\beta_{2,u}\right\}$. The parameters are estimated by nonlinear regression, with the constraint that 1 < $\;\beta_{2}\;$< 2 [56,57]. For the loading segment, the *x*-axis offset parameter estimates the zero contact point *h*_0_, while for the unloading segment, it estimates the residual indentation depth *h_p_*.

**Figure 1:**
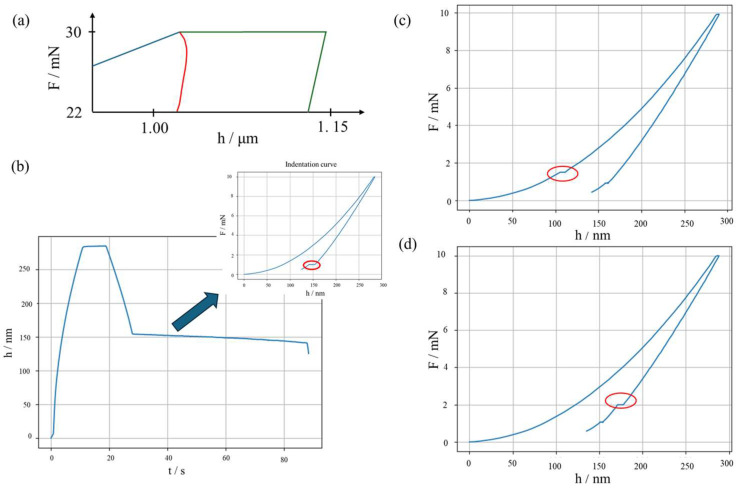
Typical measurement errors in nanoindentation. (**a**) “Nose” due to too short holding: blue, loading; red, unloading with nose; green, primary holding and unloading with correct shape. (**b**) Thermal drift: notice the slope in the secondary holding of *h*(*t*); the inset shows the longer secondary holding (circled in red), compared to a typical IC of Figure 2. (**c**) Pop-in event, highlighted by a red circle. (**d**) Pop-out event, highlighted by a red circle.

Although the system is calibrated, several measurement errors can still be introduced during the experimental procedure. First, the sample material creep response shall be compensated to avoid any unwanted dynamic contribution. This is obtained by a suitably long primary holding at the maximum force *F_max_*. If the holding is insufficient, i.e., in the presence of a significant room temperature creep, a “nose” at the onset of the unloading, see Figure 1a, can be appreciated, hindering proper evaluation of the sample contact stiffness. Second, a thermal drift is generated between the indenter tip and the sample. This generates a trend in *h* as a function of time *t*, biasing the results. The thermal drift can be compensated by introducing a secondary holding, conventionally set a 10% of *F_max_*. Such secondary holding allows estimating the slope of *h*(*t*), which is then used to correct the penetration depth measurements. Figure 1b shows examples of significant thermal drifts. Last, discontinuities in the IC can indicate an abrupt change in the material behaviour, which can be attributed to phase transformation or cracking, as shown in Figure 1c,d.

**Figure 2:**
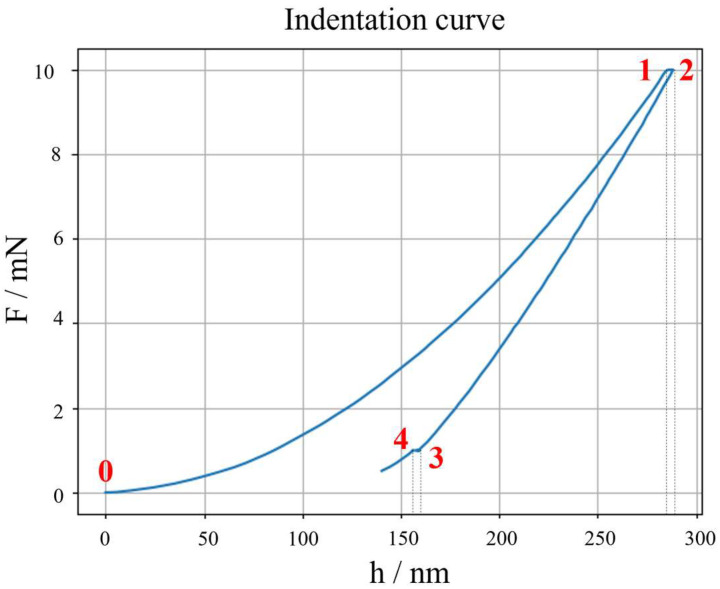
Annotation of critical IC points for continuity: 0: first contact point, 1: end of loading and beginning of primary holding, 2: end of primary holding and start of unloading, 3: end of unloading and start of secondary holding, 4: end of secondary holding.

### 1.2. Applications of Synthetic Data Generation to Nanoindentation

Nanoindentation has also been the object of synthetic data generation. Several examples can be found in the literature, and, like other measurement techniques—as discussed above—the application involves both advanced characterisation and metrology.

As far as the application for advanced characterisation, these are most often coupled with ML [58]. For example, Koumoulos et al. exploited synthetic data generation by the Synthetic Minority Over-sampling Technique (SMOTE) algorithm to increase the robustness of automatic classification and identification of reinforcement fibres in carbon fibre reinforced polymers (CFRP) [59]. Giolando et al. exploited a numerical synthetic dataset generator to solve the inverse indentation problem in biological tissues. The synthetic dataset allowed increasing experimental conditions necessary to overcome the lack of uniqueness of the solution [60]. Bruno et al. used k-NN++ synthetic data generation to impute missing data to enhance a correlative microscopy study for transformation induced plasticity (TRIP) steels [61]. Mahmood and Zia trained a generative adversarial network to generate synthetic data to support the prediction of the hardness of diamond-like carbon (DLC) coatings under varying heat treatment processing conditions [62].

Widespread adoption of synthetic dataset generation can also be found for metrological applications. Some target the model optimisation for cutting-edge testing techniques, such as SPM-based nanoindentation. For example, synthetic data were used to train an ML model estimating mechanical parameters [40], which would otherwise require a complex choice of contact model. Other applications can be found for uncertainty estimation and to support calibration methods. In particular, data-driven approaches have been used to investigate the effect of sample size on the accuracy and uncertainty of the calibration of the area shape function parameters and of the frame compliance. They were based on Monte Carlo sampling [63] and on bootstrapping [64]. The generation of synthetic data allowed highlighting a significant contribution of the calibration dataset [64] and a relevant sensitivity of calibration methods to the dataset and experimental conditions [65]. However, although practical, the bootstrapping features a severe limitation in extrapolation and prediction behaviour. Similarly, the Monte Carlo method requires obtaining accurate predictions and avoiding significant overestimation of the measurement uncertainty to model the covariance of simulated quantities, i.e., *F*(*h*).

### 1.3. Scope of the Work

This work aims to develop a traceable synthetic dataset generator for quasi-static room-temperature nanoindentation that can account for the correlation and covariance of simulated quantities. The synthetic dataset generator will be tested for accuracy and will enable associating uncertainty with the simulated results to enable the application within a metrological framework. The developed model will then be benchmarked with other alternatives, e.g., based on bootstrapping, to compare relative performances. Last, the synthetic dataset generator will include the possibility to simulate the most typical measurement errors. Such a feature will allow adopting the developed approach to train and validate advanced quality control tools for automatic measurement error detection in nanoindentation, e.g., a Digital Twin. Innovatively, the proposed method aims at establishing traceability for the synthetic datasets while keeping the experimental and computational cost effective.

The rest of the paper is structured as follows. Section 2 describes the modelling, highlighting methods to establish traceability and to evaluate measurement uncertainty. Section 3 will present results, which are discussed in Section 4. Finally, Section 5 draws conclusions and provides an outlook on future research.

## 2. Metrological Nanoindentation Synthetic Dataset Generator

This section describes first how a metrological synthetic dataset generator is obtained, and how the uncertainty of the generated data can be estimated. Later, it addresses how the main measurement errors can be simulated. Last, it presents validation methodologies to discuss accuracy with respect to real data, and relative performances with respect to other synthetic nanoindentation dataset generators for metrological applications.

This work proposes to leverage a hybrid approach to model the synthetic data generator. Such a choice, as briefly commented in the Introduction Section, aims to overcome the simplicity of analytical methods, which requires a strong hypothesis of ideal elastic behaviour and isotropy of the sample; the lack of uniqueness of solutions based on the inverse indentation problem, which challenges metrological applications; and the lack of coherence with the physics of the system of data-driven approaches. Accordingly, a physics-informed modelling is followed.

The statistical methodology is ultimately a parametric simulation approach, where the main simulation parameters, i.e., the distribution shape and related parameters, of measured quantities shall be defined to allow synthetic data generation. The approach proposes to estimate such parameters by exploiting experimental data, thereby allowing for the establishment of traceability for the synthetic dataset. The advantage of this approach lies in the fact that it allows for both modelling correlation between measured quantities and ensuring GUM-compliance for the sake of uncertainty evaluation [66,67,68,69].

The synthetic data generation modelling is discussed assuming the most typical choice of a force-controlled loading–holding–unloading force cycle, with a secondary holding for thermal drift compensation. In such a case, both the loading and unloading segments of the IC can be modelled as per Equation (2), while the two holding segments follow the assumption of a constant force as a function of time. Continuity is then constrained, with reference to Figure 2, such that if we consider the *i*-th indentation, it follows:

(3) $$F_{i}\left(h,\beta_{i}\right)=\left\{\begin{array}{c} F_{i,l}\left(h,\beta_{i,l}\right)=\beta_{i,0,l}{\left(h-\beta_{i,1,l}\right)}^{\beta_{i,2,l}}\;,\;\;F_{0,i}\leq F_{i,l}\leq F_{1,i}\;\;\\ F_{max,i},\;\;h_{1,i}\leq h_{i}\leq h_{2,i}\\ F_{i,u}\left(h,\beta_{i,u}\right)=\beta_{i,0,u}{\left(h-\beta_{i,1,u}\right)}^{\beta_{i,2,u}}\;,\;\;F_{2,i}\geq F_{i}\geq F_{3,i}\\ F_{hold2,i},\;\;h_{3,i}\leq h_{i}\leq h_{4,i} \end{array}\right.$$

where $F_{hold2}$ indicates the average, nominally constant, force of the secondary holding, and $h_{4,i}$ indicates the penetration depth at the end of the unloading.

### 2.1. Model Training

Model training is based on real data to guarantee traceability. In particular, we can consider a training set of *K* ICs, each consisting of *J* data collected at a certain sampling frequency; collected data are typically triplets of $\left\{F,h,t\right\}$. Each IC can be modelled with a power-law model for loading and unloading:

(4a) $${\hat{F}}_{i,l}=F_{i,l}\left(h,{\hat{\beta }}_{i,l}\right)={\hat{\beta }}_{i,0,l}{\left(h-{\hat{\beta }}_{i,1,l}\right)}^{{\hat{\beta }}_{i,2,l}},\;i:1,\ldots ,K$$

(4b) $${\hat{F}}_{i,u}=F_{i,u}\left(h,{\hat{\beta }}_{i,u}\right)={\hat{\beta }}_{i,0,u}{\left(h-{\hat{\beta }}_{i,1,u}\right)}^{\beta_{i,2,u}},\;i:1,\ldots ,K$$

Parameters ${\hat{\beta }}_{i}$ are estimated by nonlinear orthogonal distance regression (ODR) to account for error-in-variables. In fact, ODR assumes error both in the response (ε) and in the regressors (δ), e.g., $F_{i,l}(h,\beta_{i,l})=f_{i,l}\left(h+\delta_{i,l};\beta_{i,l}\right)+\varepsilon_{i,l}$, and allows obtaining an estimation of model parameters by minimising $\sum_{j=1}^{J_{l}}\varepsilon_{i,l,j}^{2}+\delta_{i,l,j}^{2}$, which are both expressed for the loading segment of the IC.

Then, it is possible to estimate the average parameters and the mean squared errors of the residuals for both the loading and unloading segments of the IC, e.g., Equations (5a), (5b) and (6) exemplify computations for the loading segment:

(5a) $$\bar{{MSE}_{F,l}}=\frac{1}{K}\sum_{i=1}^{K}{MSE}_{i,l}$$

(5b) $$MSE_{i,l}=\frac{\sum_{j=1}^{J}{\left(F_{i,l,j}-{\hat{F}}_{i,l,j}\right)}^{2}}{J}$$

(6) $${\bar{\beta_{l}}}_{K}=\frac{1}{k}\sum_{i}^{K}\beta_{i,l}$$

According to the modelling of Equation (4a) and (4b) for the loading and unloading segment of the ICs, it is also possible to invert the model to express the estimated penetration depth:

(7) $${\hat{h}}_{i}=h_{i}\left(F,{\hat{\beta }}_{i}\right)={\hat{\beta }}_{i,1}+{\left(\frac{F}{{\hat{\beta }}_{i,0}}\right)}^{\frac{1}{{\hat{\beta }}_{i,2}}}$$

### 2.2. Data Generation

For each synthetic indentation and for each segment of the indentation curve, the number of points *J* is sampled from a normal distribution with mean and variance evaluated from the *K* training curves, i.e.,

(8) $$J\sim N\left({\bar{J}}_{K},s_{K}^{2}(J)\right)$$

where the ${\bar{J}}_{K}$ indicates the sample average of *K* observations, and $s_{K}^{2}$ indicates the sample variance of *K* observations.

Then, for each synthetic indentation and for each segment of the indentation curve, the start time, as in Equation (9a), and end time, as in Equation (9b), are sampled from a normal distribution with mean and variance evaluated from the *K* curves to train the model. The time vector ***t***, see Equation (9d), is then simulated as a linear spacing of *J* data in the overall duration ∆t, as in Equation (9c). The variance of the nanoindentation experiment overall duration is obtained as $s_{K}^{2}\left(∆t\right)=s_{K}^{2}\left(t_{start}\right)+s_{K}^{2}\left(t_{end}\right)$.

(9a) $$t_{start}\sim N\left({\bar{t_{start}}}_{K},s_{K}^{2}\left(t_{start}\right)\right)$$

(9b) $$t_{end}\sim N\left({\bar{t_{end}}}_{K},s_{K}^{2}(t_{end})\right)$$

(9c) $$∆t=t_{end}-t_{start}\sim N\left({\bar{∆t}}_{K},s_{K}^{2}\left(∆t\right)\right)$$

(9d) $$t=\left\{0:\frac{∆t}{J}:∆t\right\}$$

#### 2.2.1. Loading Segment Generation

With reference to Figure 2, the force at point 0, *F*_0_, is sampled from a normal distribution with mean and variance evaluated from the *K* training ICs, see Equation (10), and the force at point 1, *F*_1_, is sampled from a normal distribution with mean and variance from the primary holding of the *K* experimental curves, as in Equation (11).

(10) $$F_{0}\sim N\left({\bar{F_{0}}}_{K},s_{K}^{2}(F_{0})\right)$$

(11) $$F_{1}\sim N\left({\bar{F_{1}}}_{K},s_{K}^{2}(F_{1})\right)$$

Model parameters $\beta_{l}$ are sampled considering that the estimates by the regression come from a multivariate normal distribution with mean ${\bar{\beta_{l}}}_{K}$ and covariance matrix ${\bar{\Sigma_{\beta ,l}}}_{K}$, as in Equation (12):

(12) $$\beta_{l}\sim N\left({\bar{\beta_{l}}}_{K},{\bar{\Sigma_{\beta ,l}}}_{K}\right)$$

Then, the penetration depth at point 1, $h_{1}$, is evaluated as in Equation (13).

(13) $$h_{1}=h\left(F_{1},\beta_{l}\right)=\beta_{1,l}+{\left(\frac{F_{1}}{\beta_{0,l}}\right)}^{\frac{1}{\beta_{2,l}}}$$

Since a force-controlled cycle is considered, the force vector for the loading segment ***F_l_*** is simulated as a linear spacing vector between *F*_0_ and *F*_1_, as in Equation (14a). A point-wise zero-mean random noise is then added to cater for measurement noise, as in Equation (14b)

(14a) $$F_{l}=\bar{F_{l}}+e_{F,l}=\left\{F_{0}:\frac{F_{1}-F_{0}}{J_{l}}:F_{1}\right\}+e_{F,l}$$

(14b) $$e_{F,l}\sim N\left(0,\bar{{MSE}_{F,l}}\right)$$

Then, the penetration depth vector is simulated from the regression curve $h_{l}\left(\bar{F_{l}},\beta_{l}\right)$, as described in Equation (7), constraining $h_{l}\in [h_{0};h_{1}]$. No further measurement noise, simulating reproducibility, is added as it is already included in the distributions of $F_{l}$ and $\beta_{l}$.

#### 2.2.2. Primary Holding Generation

The primary holding is characterised by the maximum applied force *F_max_* and by the duration. The primary holding aims to compensate for the room-temperature creep. In fact, the control parameters (*F_max_* and duration) induce an increase in the penetration depth Δh computed as the difference between the first and last point of the primary holding, i.e., $\Delta h=h_{2}-h_{1}$, defined with reference to Figure 2. Δh is sampled from a normal distribution with mean and variance evaluated from the *K* training ICs as in Equation (15), where the variance is obtained by combining individual contributions as $s_{K}^{2}\left(∆h\right)=s_{K}^{2}\left(h_{2}\right)+s_{K}^{2}(h_{1})$.

(15) $$\Delta h\sim N\left({\bar{\Delta h}}_{K},s_{K}^{2}(∆h)\right)$$

Then, from the previously sampled $h_{1}$, as per Equation (13), the penetration depth at point 2, $h_{2}$, is evaluated as in Equation (16a). The penetration depth vector for the primary holding, $h_{holding1}$, is then simulated as a linear spacing vector between $h_{1}$ and $h_{2}$, as in Equation (16b). A point-wise random noise is added to account for the accuracy, assumed normally distributed, with zero mean, and variance proportional to the measured penetration depth, see Equation (16c) [42]. No measurement reproducibility is further added because it is already included in the distributions of $h_{1}$ and ∆h.

(16a) $$h_{2}=h_{1}+∆h$$

(16b) $$h_{holding1}=\left\{h_{1}:J_{holding1}:h_{2}\right\}+{Acc}_{h}$$

(16c) $${Acc}_{h}\sim N\left(0,{\left(h\cdot u_{Acc,h,\%}\right)}^{2}\right)$$

The associated force vector, $F_{holding1}$, is simulated, as in Equation (17a), as a constant force value equal to $F_{1}$ (with $F_{1}$ = $F_{2}$), sampled from Equation (11). A point-wise random noise is added to account for the assumed normally distributed, with zero mean, and variance proportional to the measured force, see Equation (17b) [42]. No measurement reproducibility is further added because it is already included in the distributions of $F_{1}$.

(17a) $$F_{holding1}=\left\{F_{1}:J_{holding1}:F_{2}\right\}+{Acc}_{F}$$

(17b) $${Acc}_{F}\sim N\left(0,{\left(F\cdot u_{Acc,F,\%}\right)}^{2}\right)$$

#### 2.2.3. Unloading Generation

The process is highly similar to the generation of the loading, described in Section 2.2.1. The parameters $\beta_{u}$ of the regression are sampled from a multivariate normal distribution with mean ${\bar{\beta_{u}}}_{K}$ and covariance matrix ${\bar{\Sigma_{\beta ,u}}}_{K}$, both evaluated from the *K* training ICs, i.e., similarly to Equation (12) $\beta_{u}\sim N\left({\bar{\beta_{u}}}_{K},{\bar{\Sigma_{\beta ,u}}}_{K}\right)$. The force at point 3, with reference to Figure 2, is sampled from a normal distribution with mean and variance from the secondary holding, empirically estimated, as in Equation (18), which allows estimating the penetration depth at point 3, $h_{3}$, by inverting the regression model, as in Equation (19).

(18) $$F_{3}\sim N\left({\bar{F_{3}}}_{K},s_{K}^{2}(F_{3})\right)$$

(19) $$h_{3}=h\left(F_{3},\beta_{u}\right)=\beta_{1,u}+{\left(\frac{F_{3}}{\beta_{0,u}}\right)}^{\frac{1}{\beta_{2,u}}}$$

Then, the force vector $F_{u}$ is simulated as a linear spacing vector between $F_{2}$(= $F_{1}$) and $F_{3}$, as described by Equation (20a). A point-wise random noise is added, assuming a zero mean normal distribution with the variance estimated as in Equation (20b).

(20a) $$F_{u}=\bar{F_{u}}+e_{F,u}=\left\{F_{2}:\frac{F_{3}-F_{2}}{J_{u}}:F_{3}\right\}+e_{F,u}$$

(20b) $$e_{F,u}\sim N\left(0,\bar{{MSE}_{F,u}}\right)$$

Finally, the penetration depth vector is simulated from the regression curve $h_{u}\left(\bar{F_{u}},\beta_{u}\right)$, as described in Equation (7), constraining $h_{u}\in [h_{2};h_{3}]$. No further measurement noise, simulating reproducibility, is added as it is already included in the distributions of $F_{u}$ and $\beta_{u}$.

#### 2.2.4. Secondary Holding Generation

Also in this case, the generation process is highly similar to the one introduced in Section 2.2.2 for the primary holding. The secondary holding is introduced to compensate for thermal drifts, which will be simulated in Section 2.4. Conversely, in nominal conditions, a constant force and penetration should be obtained.

Accordingly, the penetration depth vector is created as a constant value equal to $h_{3}$ (with $h_{4}$ = $h_{3}$), as described in Equation (21), to which a point-wise random noise is added to account for the measurement accuracy.

(21) $$h_{holding2}=\left\{h_{3}:J_{holding2}:h_{4}\right\}+{Acc}_{h}$$

The force vector is created as a constant force value equal to $F_{3}$ (with $F_{4}$ = $F_{3}$), to which point-wise random noise is added to account for the accuracy, as in Equation (22)

(22) $$F_{holding2}=\left\{F_{3}:J_{holding2}:F_{4}\right\}+{Acc}_{F}$$

### 2.3. Uncertainty Evaluation

Synthetically generated quantities are obtained by sampling from underlying statistical distributions. Accordingly, it is possible to estimate the uncertainty of the synthetic indentation curve. The loading and unloading segment uncertainty evaluation leverages the law of propagation of uncertainty (LPU) [70] and caters for the fact that model parameters have been estimated by ODR. According to the LPU, the combined variance is obtained as a linear combination of the variance contributions weighted for the squared sensitivity coefficients, i.e., the partial derivatives of the response, in this case *F*, to the independent quantities. The model for partial derivative estimation is defined in Equation (23), e.g., for the loading segment.

(23) $$F_{l}\left(h,\beta_{l}\right)=f_{l}\left(h\pm \delta_{l};\beta_{l}\right)\pm e_{F,l}=\beta_{0,l}{\left(h\pm \delta_{l}-\beta_{1,l}\right)}^{\beta_{2,l}}\pm e_{F,l}$$

In Equation (23), $\delta \sim N\left(0,ms\delta \right)$ estimates the error in the regressor variable, and $e_{F}$, as introduced in Equations (14b) and (20b), describes the residual error. In particular, on the regression residuals, e.g., for the loading segment as in Equation (23), it is possible to evaluate $ms\delta_{l}=\frac{1}{J_{l}}\sum_{i=1}^{J_{l}}\delta_{i,l}^{2}$. The *mse* includes, by definition, the error in the response ε, which is also subject to minimization for estimating the parameters β by ODR.

Applying the LPU to the metrological model of Equation (23), the variance of the simulated force can be obtained as in Equation (24), which is written, for example, for the loading segment.

(24) $${u^{2}}_{F_{syntehtic}}={u^{2}}_{F,l}={\left[\begin{array}{c} \frac{\partial F}{\partial h}\\ \frac{\partial F}{\partial \delta }\\ \frac{\partial F}{\partial \beta }\\ \frac{\partial F}{\partial e_{F}} \end{array}\right]}^{T}\left[\begin{array}{cccc} {u^{2}}_{h} & 0 & 0 & 0\\ 0 & ms\delta_{l} & 0 & 0\\ 0 & 0 & {\bar{\Sigma_{\beta_{l}}}}_{K} & 0\\ 0 & 0 & 0 & {u^{2}}_{F}+mse_{l} \end{array}\right]\left[\begin{array}{c} \frac{\partial F}{\partial h}\\ \frac{\partial F}{\partial \delta }\\ \frac{\partial F}{\partial \beta }\\ \frac{\partial F}{\partial e_{F}} \end{array}\right]$$

In Equation (24), $u_{h}$ and $u_{F}$ are the standard uncertainties of the displacement sensor and the force transducer, typically obtained from a calibration certificate, which shall be added to include contributions from the traceability chain. ${\bar{\Sigma_{\beta }}}_{K}$ is the already introduced covariance matrix of the model parameters, estimated by ODR, which caters for the uncertainty in the parameters estimation.

### 2.4. Error Simulation Approaches

The metrological synthetic dataset generator for nanoindentation described in Section 2.2 also aims at generating the most typical measurement errors. Simulating errors is useful to allow training of error detection models, as reviewed in Section 1.2. In this work, three main errors are modelled: thermal drift, pop-in, and pop-out.

#### 2.4.1. Thermal Drift Simulation

Even in controlled laboratory conditions, a thermal gradient can be present between the indenter tip and the sample. This can occur due to insufficient stabilisation of the sample, because of electronics heating the indenter through conduction, and because of the small amount of heat dissipated through friction during the indentation. To evaluate the thermal drift q(T), the most robust approach, even though not the most time efficient, consists of performing a secondary holding and evaluating the slope of *h*(*t*), i.e., $q\left(T\right)={\left.\frac{dh}{dt}\right|}_{holding2}$, such that: $h_{holding2}=h_{a}+q(T)t$ [42,71]. In ideal conditions, q(T)=0. However, this is never the case. In optimal experimental conditions, i.e., after a long thermal stabilisation of the sample in the measurement environment, the heating due to electronics plays a major role, typically inducing a thermal flux from the indenter to the sample, inducing q(T)<0 [41,51].

Presence of thermal drift can be simulated by modifying all the penetration depths generated in Section 2.2, such that

(25) $$h_{j}=h_{j}+q(T)\cdot \left(t_{j}-t_{0}\right)$$

Provided that any thermal drift can be generated, if a real-world scenario traceable to experimental data is aimed at, q(T) can be sampled from the *K* training ICs, assuming a normal distribution $q(T)\sim N\left({\bar{q\left(T\right)}}_{K},s_{K}^{2}\left(q(T)\right)\right)$.

#### 2.4.2. Pop-In Simulation

A pop-in event describes a singularity in the loading segment, such that at a given force level, a discontinuity $∆h_{pop-in}$ in the penetration depth occurs, suddenly increasing the penetration depth. This phenomenon is typically associated with phase changes, e.g., in semiconductors [72,73], or cracking, e.g., for coatings [74]. Accordingly, a pop-in event can be simulated by adding from a certain time instant onwards, during the loading segment, a shift in the penetration depth simulated, according to Section 2.2.1, i.e., $h\left(t>t^{*}\right)=h\left(t\right)+∆h_{pop-in}$, with $t^{*}\in \left[t_{0};t_{1}\right]$. The specific selection of the $t^{*}$ that induces the pop-in event shall be modelled depending on the material under study considering the specific loading cycle. In this work, to demonstrate capability of the synthetic dataset generator to include pop-in events, $t^{*}$ was randomly selected between the instants realising $F\in [F_{0};30\%F_{max}]$ during the loading.

#### 2.4.3. Pop-Out Simulation

Quite in a dual manner, a pop-out event describes a sudden decrease in the penetration depth. Pop-out is typically induced by phase transformation to metastable phases [72,75], or by cracks closing [73], and is induced by the load removal. Thus, it typically takes place during the final part of the unloading.

Accordingly, a pop-out event can be simulated by removing from a certain time instant onwards, during the unloading segment, a shift in the penetration depth simulated, according to Section 2.2.3, i.e., $h\left(t>t^{*}\right)=h\left(t\right)-∆h_{pop-out}$, with $t^{*}\in \left[t_{3};t_{J_{4}}\right]$. Also for pop-out, the selection of $t^{*}$ is strictly material dependent. Thus, to simply demonstrate capability of the synthetic dataset generator to include pop-out events, $t^{*}$ was randomly selected between the instants realising $F\in [10\%;30\%]F_{max}$ during the unloading.

### 2.5. Validation Methodology

The validation of the synthetic dataset generator is performed by testing its capability of simulating data from the real world. In particular, an Anton Paar STeP6 platform equipped with an NHT^3^ nanoindentation head is considered. The equipment, hosted in the metrological laboratory of the MInd4Lab of Department of Management and Production Engineering of Politecnico di Torino, was equipped with a Berkovich indenter, calibrated [63] and used to perform a set of 15 indentations of a certified reference material, i.e., a NPL-calibrated sample of SiO_2_, having the Young modulus of (73.0 ± 0.5) GPa and Poisson’s ratio of 0.163 ± 0.002. Indentations implemented a force-controlled indentation cycle with a maximum force of 10 mN, and duration of loading, primary holding, and secondary holding (at 10% of *F_max_*), respectively, of 10 s, 8 s, 9 s, and 60 s. The force and displacement sensors have, respectively, a calibrated relative accuracy of 0.061% and 0.058%. Raw force and displacement data, i.e., not automatically corrected for frame compliance, are considered to apply the full analysis pipeline described in previous sections.

The validation aims to test that real-world data cannot be statistically distinguished from the synthetically generated dataset, with a confidence level of 95%. That is, a hypothesis test based on the t-Student distribution, with a null hypothesis $H_{0}:F_{synthetic}=F_{experimental}$ is built, having the test statistic $t_{stat}=\frac{F_{synthetic}-F_{experimental}}{u_{F_{synthetic}}}$. The test statistic estimates the standard uncertainty of the synthetically generated force as $u_{F_{synthetic}}$, according to Equation (24). Since Equation (24) includes traceability and reproducibility through $u_{F}$ and the *mse,* to avoid overestimation, it is assumed that the dispersion to experimental data is already included.

Further validation is performed by comparing the performances of the proposed metrological synthetic data generator for nanoindentation based on parametric simulation with other approaches available in the literature. In particular, a simulation based on non-parametric bootstrapping is considered. The methodology for bootstrap generation is reported elsewhere [64], and it has been proven effective in various metrological applications [65] overcoming the limits of Monte Carlo approaches. In particular, the presence of significant bias is tested, as well as any effect of systematic underestimation of measurement uncertainty. The former is once more tested by means of a t-Student hypothesis test. The latter by means of a hypothesis test based on the F-Fisher distribution, having the null hypothesis $H_{0}:u_{F_{synthetic}}^{2}=u_{F_{Bootstrap}}^{2}$, and test statistic $F_{stat}=\frac{u_{F_{synthetic}}^{2}}{u_{F_{Bootstrap}}^{2}}$.

## 3. Results

The collected data, as per Section 2.5, were used to establish a traceable synthetic dataset generator for nanoindentation following the methodology described in Section 2.1.

**Figure 3:**
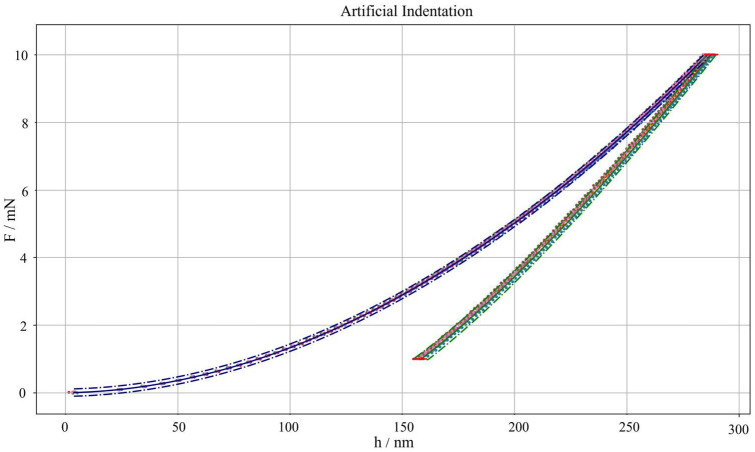
IC of synthetically generated nanoindentations. Points represent synthetically generated curves; lines are the theoretical model from experimental data. Blue: loading segment, green: unloading segment, red: holding segments. Solid lines are average predictions, and dot-dashed lines represent prediction uncertainty intervals with a confidence interval of 95%.

**Figure 4:**
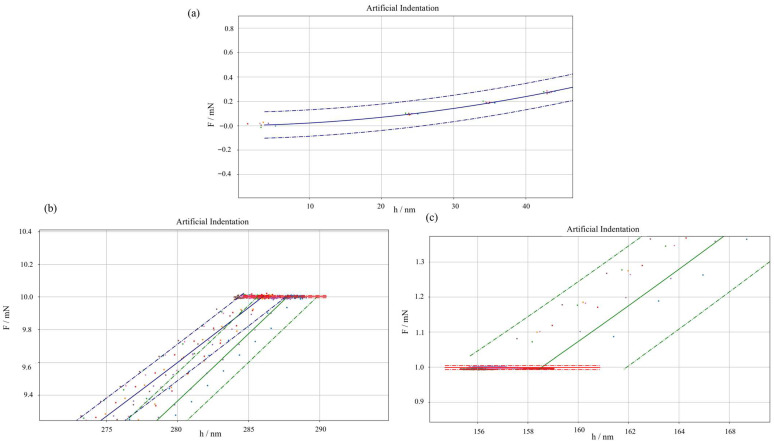
Insights into synthetically generated nanoindentation curves. (**a**) Start of indentation and zero point, (**b**) primary holding, and (**c**) secondary holding. Points represent synthetically generated curves; lines are the theoretical model from experimental data. Solid lines are average predictions, and dot-dashed lines represent prediction uncertainty intervals with a confidence interval of 95%.

Figure 3 shows the results of the application of the metrological synthetic data generator according to the methodology outlined in Section 2.2. Insights into relevant and critical regions of the synthetically generated curves are shown in Figure 4. Figure 3 and Figure 4 show the reference model with associated uncertainty with a 95% confidence interval, evaluated as per Section 2.3.

### 3.1. Generation of Errors

**Figure 5:**
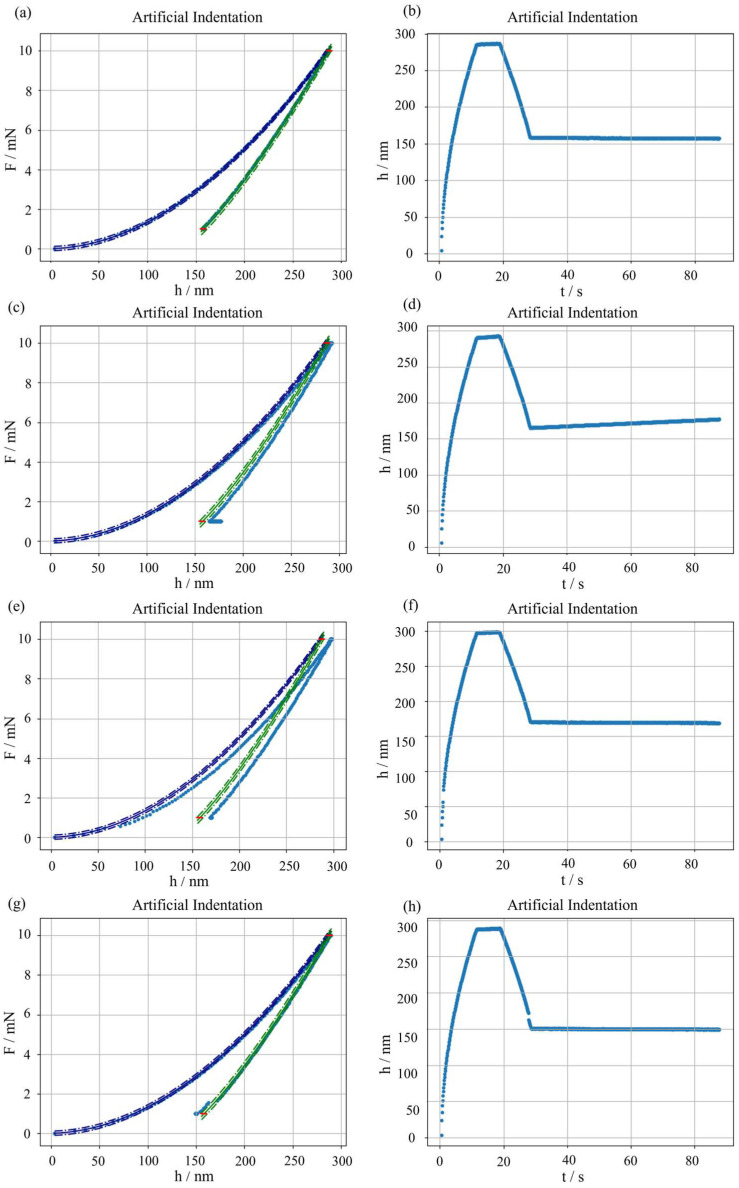
Simulation of main measurement errors in the synthetic dataset. No error (typical measurement): (**a**) IC, (**b**) penetration depth as a function of time. Significant thermal drift: (**c**) IC, (**d**) penetration depth as a function of time. Pop-in: (**e**) IC, (**f**) penetration depth as a function of time. Pop-out: (**g**) IC, (**h**) penetration depth as a function of time.

The traceable synthetic dataset generator for nanoindentation is then applied to simulate main measurement errors, to test the methodology described in Section 2.4. Figure 5c–f shows the successful application of the proposed parametric generation approach with examples for a significant thermal drift, pop-in, and pop-out, while Figure 5a,b show, for reference, a typical IC and *h*(*t*).

As far as the thermal drift is concerned, a relevant slope is introduced, see Figure 5d, which induces an overall distortion of the whole IC, as well as a more apparent secondary holding, see Figure 5c.

Regarding discontinuities, pop-in and pop-out are simulated effectively. Figure 5e shows a pop-in in the loading segment, also clearly visible in the lack of continuity of the related *h*(*t*) in Figure 5f. The pop-in offsets the subsequent points of the IC of a constant value, as shown in Figure 5e. Similarly, Figure 5g shows a pop-out in the unloading segment, also clearly visible in the lack of continuity of the related *h*(*t*) in Figure 5h.

### 3.2. Validation

Following the methodology introduced in Section 2.5, the validation of the metrological synthetic dataset generator is performed.

First, the accuracy of the synthetic dataset is assessed by performing a t-Student hypothesis test to compare the synthetic data with experimental data. A graphical representation can be obtained by checking that synthetic datapoints all fall inside the prediction intervals, i.e., the dot-dashed lines in Figure 3. As can be appreciated, the null hypothesis cannot be rejected, with a risk of error of 5%, successfully validating the synthetic dataset generator accuracy. More in detail, insights from Figure 4 show that only a few points (less than 0.1% of generated points), near the transition to one segment to another, are outside the confidence interval limits. This is consistent with how measurement noise was introduced in the synthetic dataset generator to perturb the system further.

**Figure 6:**
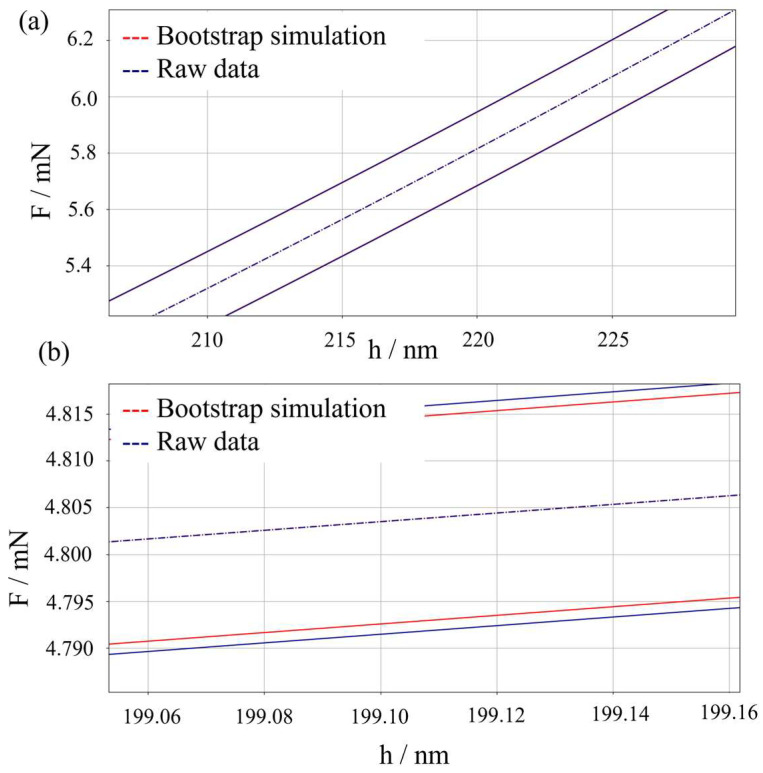
Effect of bootstrapping on measurement uncertainty. Prediction intervals at a 95% confidence level with two different levels of magnification (**a**,**b**) to appreciate the difference. Red: bootstrapping method, blue: parametric synthetic data generator based on raw data.

Lastly, a comparison with respect to a non-parametric synthetic dataset generation approach based on bootstrap [64] is performed. Figure 6 shows an inset of the loading segment of the IC, highlighting the effect of the synthetic dataset generator. As can be appreciated, the method proposed in this work, i.e., a parametric synthetic dataset generator, produces a slight overestimation of the measurement uncertainty. However, when the statistical significance of such overestimation is tested by means of a hypothesis test based on the F-Fisher distribution, a *p*-value of 12% results, showing that such overestimation is not statistically significant. As can be appreciated, no systematic shift in the mean prediction can be seen.

## 4. Discussion

The methodology outlined in Section 2 ultimately consists of a parametric synthetic dataset generator for nanoindentation.

With respect to other parametric approaches [63], it allows modelling the correlation between input quantities, thus overcoming possible distortions in the uncertainty evaluation.

Similarly, with respect to non-parametric methods, i.e., based on bootstrapping [64], it has shown no systematic differences, neither in terms of mean nor of dispersion. Compared to bootstrapping, it requires less computational power, is much faster, and allows overcoming issues of sample representativeness needed to perform the non-parametric approach.

The method requires modelling the indentation response of a material by means of a power-law model. Although the mathematical implementation allows for any set of parameters to be used, without the need for an experimental training dataset, the suggested approach in Section 2.1 allows, by means of Orthogonal Distance Regression, to establish a traceable and physics-informed model. Indeed, when the regression is performed to evaluate model parameters, the model representativeness is limited to a specific combination of material and indentation machine. In such a case, the proposed method limits the representativeness of the model to a specific material and specific indentation cycle parameters, i.e., the maximum force and indentation cycle segments’ durations. Such limitation is inherent with the proposed methodology and can be considered the cost for having a traceable synthetic dataset generator. To extend the validity of the model to other materials and indentation machines, being the response intertwined due to the indenter geometry and frame compliance, a new training dataset is required.

Further, an underlying assumption of the synthetic dataset generator is the requirement of homogeneous material for training data. This condition is easily met for amorphous materials and for monocrystals. Conversely, for multiphase materials and polycrystalline materials, if the indentation scale of interest allows to distinguish different phases, a separate synthetic dataset generator per each phase can be trained relying on phase-specific data.

## 5. Conclusions

This work has proposed a parametric metrological synthetic dataset generator for quasi-static room-temperature nanoindentation. By means of statistical modelling, the proposed approach allows catering for the main uncertainty source, i.e., traceability and reproducibility, in the data generation process, thus enabling a metrological dataset generation. The accuracy of the method has been successfully tested against real-world data.

Although limited to specific experimental conditions, the synthetic dataset generator proposed in this work can be used to generate a traceable dataset. Future work will test the applicability of the method on multiphase materials and coatings. Traceable datasets generated by the synthetic dataset generator here proposed will find application in future works aiming to relieve the experimental effort needed to collect an extensive dataset for training more flexible simulative systems based on finite element simulation, e.g., based on inverse indentation problem, by providing a traceable reference dataset for validating advanced measurement quality control tools based on Digital Twins.

## Data Availability

The data used in this study and the code for the Synthetic dataset generation according to the proposed methodology are available on Zenodo at 10.5281/zenodo.17465545.

## Abbreviations

The following abbreviations are used in this manuscript:

AI	Artificial Intelligence
DT	Digital Twin
*F*	Measured applied force
GUM	Guide to the expression of Uncertainty in Measurement
*h*	Measured penetration depth
*J*	Number of data points in each nanoindentation
*K*	Number of experimental nanoindentations
IC	Indentation Curve
ML	Machine Learning
*t*	Measured time
